# P18 Prevalence and risk factors for nasal colonization with MRSA among children attending immunization in Port Harcourt, Nigeria

**DOI:** 10.1093/jacamr/dlac004.017

**Published:** 2022-02-16

**Authors:** Chinekwu N. Oreh

**Affiliations:** Faculty of Pharmaceutical Sciences, University of Port Harcourt, Nigeria; Nigeria Governors Forum Secretariat, Nigeria

## Abstract

**Background:**

*Staphylococcus aureus* is a bacterial agent commonly found on the skin and anterior nares of healthy individuals. There has been an increase in the carriage of MRSA globally. Nasal MRSA colonization is a public health problem because it is a significant risk factor for staphylococcal infection. This study explores the prevalence, antibiotic susceptibility profile and risk factors for nasal colonization of healthy children under 5 years old attending immunization.

**Methods:**

Nasal swabs were collected from 176 healthy children 0–60 months old, attending routine immunization in Port Harcourt. Questionnaires covering demographic information and potential risk factors were administered to caregivers of the children. Collected samples were tested for the presence of MSSA and MRSA. Antibiotic susceptibility testing was carried out on all MSSA and MRSA samples.

**Results:**

The prevalence of MSSA and MRSA were 10/176 (5.68%) and 48/176 (27.3%), respectively. The MDR rate among the 58 *S. aureus* samples was 53/58 (91.38%). Children who had an ear infection in the preceding 1 month were at a higher risk of being colonized with MRSA at *P = *0.048 (95% CI). There was also a significant association *P = *0.036 (95% CI) between the educational status of mother and nasal colonization, with higher educational status reducing the chances of association.

**Conclusions:**

There is a rising prevalence of MRSA colonization and MDR strains. A history of ear infections and a poor level of education of the mother were identified as significant risk factors for MRSA colonization.

**Table 1.** Association between potential risk factors and MRSA carriage

Factors	MRSA		χ^2^	*P* value
	positive	negative		
Hospitalization			0.377	0.539*^f^*
Yes	0	1		
No	48	127		
Hospital visit			0.140	0.708
Yes	5	11		
No	43	117		
RTI			0.863	0.353
Yes	24	74		
No	24	54		
Ear infection			4.860	0.048* *^f^*
Yes	4	2		
No	44	126		
Antibiotic use			1.375	0.241
Yes	9	35		
No	39	93		
Family health worker			0.304	0.582
Yes	5	10		
No	43	118		
Smoker			1.638	0.243*^f^*
Yes	2	13		
No	46	114		

* Significant at 95%; *^f^*Fisher's exact test.

